# Attitudes of support people: a key element when implementing technologies for people with intellectual and visual disabilities

**DOI:** 10.1080/17483107.2024.2387774

**Published:** 2024-08-08

**Authors:** Lotte Piekema, Annet ten Brug, Aly Waninge, Annette van der Putten

**Affiliations:** a Unit of Inclusive and Special Needs Education, University of Groningen, Groningen, the Netherlands; b Research Group Healthy Ageing, Hanze University of Applied Sciences Groningen, Groningen, the Netherlands; c University Medical Center Groningen, Health Psychology Research, University of Groningen, Groningen, the Netherlands

**Keywords:** Intellectual disabilities, support needs, technology, effort expectancy, attitude, behavioural intention, implementation

## Abstract

**Aim:**

The degree to which people with intellectual and visual disabilities can use technology relies on the level of support they receive. However, there is a lack of knowledge about the relationship between the constructs effort expectancy, attitudes, and behavioural intentions of support people (i.e. relatives and healthcare professionals) regarding the use of such technologies for people with intellectual and visual disabilities. The aim of this study was to gain insight into how these constructs are connected and to explore their relationship with support person’s characteristics and the support need levels of people with intellectual and visual disabilities.

**Methods:**

In total, 186 support people from a Dutch healthcare organisation focusing on people with intellectual and visual disabilities participated in an online questionnaire. We used a regression analysis to explore how the constructs, the characteristics, and the level of support needs were related.

**Results:**

Both effort expectancy (β = .35; t(185) = 5.04; p < .001) and attitude (β = .75; t(185) = 15.55; p < .001) of support people were related to the intention to use technologies. The effect of effort expectancy (β = .04; t(177) = .74; p = .462) on the intention to use technologies was mediated through attitude (β = .74; t(177) = 13.28; p < .001). Younger support people scored higher on attitude than older support people.

**Conclusions:**

Support people’s effort expectancy and attitude play a significant role in their intention to use technologies when supporting people with intellectual and visual disabilities, with attitude emerging as pivotal factor.

## IMPLICATIONS FOR REHABILITATION

- A better understanding of the relationship between effort expectancy, attitude, and the intention to use technologies in support people may facilitate the implementation of technologies.
- The intentions of support people to use technologies is not related to the level of support needs of people with intellectual and visual disabilities.
- Younger support people have a more positive attitude towards technology for people with support needs compared to older support people.
- A positive attitude has a strong and positive relationship to a higher behavioural intention. Therefore, when implementing technologies, healthcare organisations should pay special attention to the attitude of support people towards the use of technologies for people with support needs.

## Introduction

People with intellectual and visual disabilities can use technologies, but they may need support when doing so. An intellectual disability is characterised by a limitation in intellectual functioning and in adaptive behaviour [1]. The prevalence of visual impairments and blindness is higher in people with intellectual disabilities, with an elevated prevalence in people with more severe intellectual disabilities [2–4]. People with intellectual and visual disabilities form a very diverse group and their support needs can range from intermittent to pervasive care, depending on the severity of the intellectual disability, visual disability, motor disabilities, and the presence of concomitant disabilities [1,5]. People with profound intellectual and multiple disabilities have high and specialised support needs in all daily living situations [1,5,6], including when using technology. People with mild intellectual disabilities often have fewer support needs [1,7] and are therefore considered to be more independent when using technologies. However, other challenges, such as privacy, may play a role when people with mild intellectual disabilities use technologies [7].

The successful adoption of any intervention depends on stakeholder willingness and the perceived ease of the application, and thus the people involved will play an important role in determining implementation success [8,9]. These stakeholders, which are mostly relatives or healthcare professionals, usually provide assistance. Although relatives are mostly responsible for the care of the person with intellectual and visual disabilities, know the person’s needs and preferences, and provide life-long care, providing this care in collaboration with healthcare professionals improves the well-being of both the person with intellectual and visual disabilities as well as the relatives [10,11]. Involving both relatives and healthcare professionals may also be important when new technologies are implemented in the support of people with intellectual and visual disabilities. These technologies may be targeted to assist the person with intellectual and visual disabilities directly or to improve the context of their care. For example, a sensor sock that measures physiological signals such as heart rate and skin conductance of a person with intellectual and visual disabilities can help support people to understand pain signals and respond to these signals accordingly [12]. In addition, smart continence care can help reduce the number of leakages and changes [13].

There are different types of technologies that are used in the support of people with intellectual and visual disabilities, including mainstream technologies (MT) or commercial devices, such as tablets and game consoles, or assistive technologies (AT), such as augmentative communication aids and eye trackers [14–16]. Technologies can also be designed with the principles of universal design (UD) in mind and be more accessible to everyone, such as wearables and smart home solutions [16,17].

Technology can be beneficial in the daily lives of people with intellectual and visual disabilities and can have a positive effect in both people with fewer support needs, such as those with mild intellectual disabilities [18,19], and in people with intensive and specialised support needs, such as those with profound intellectual and multiple disabilities [7,20]. For example, technologies might increase engagement in activities, psychological well-being, and social participation in people with disabilities [21]. A study by [22] showed that Information and Communication Technology (ICT) can help people with mild to moderate intellectual disabilities to be more independent and engage in social participation and leisure activities. Furthermore, the use of e-learning has been found to improve social, decision-making, and communication skills in people with intellectual disabilities [23], as well as to enhance interpersonal relationships and self-determination in people with profound intellectual and multiple disabilities [20].

It is crucial to focus on the implementation process when using technologies aimed to assist people with intellectual and visual disabilities, because successful use of technological interventions is related to appropriate implementation strategies [24,25]. The opportunity to use technologies to assist people with intellectual and visual disabilities also depends in varying degrees on support people. Therefore, when implementing such technologies, the focus should also be on the support person. Several models aim to explain the process of implementation of interventions, one of which is specifically aimed at technology-based interventions: the Unified Theory of Acceptance and Use of Technology (UTAUT) [8]. This theory-based model aims to understand a support person’s level of acceptance of technologies by measuring the support person’s behavioural intention. Behavioural intention is associated with actual use of technologies in practice, which suggests that higher behavioural intention is related to more use of technologies.

The UTAUT describes four constructs related to behavioural intention [8]: performance expectancy, effort expectancy, social influence, and facilitating conditions. Performance expectancy concerns a person’s belief that the use of technology will enhance performance. Effort expectancy is the degree of ease anticipated in using technologies. Social influence is how a person perceives the opinion of significant others and is related to subjective norms, social factors, and image [8]. The fourth construct, facilitating conditions, concerns whether a person believes that they are supported on the organisational and technological levels when using technologies.

In the general population, the effects of these constructs on behavioural intention of the person using the technology are influenced by gender, age, experience, and voluntariness of use [8], where, for example, effort expectancy tends to be greater in women, older people, people with less experience, and when something is compulsory. In addition, the intention to use technologies might also be related to the intensity of support needs, while the intention to use technology might be less obvious in people with severe to profound intellectual disabilities [7].

Attitude may be considered as the way a person feels about technologies and their degree of interest in technologies. A multifaceted process involving cognitive, affective, and behavioural aspects may influence the formation and expression of an attitude [26]. Earlier studies have observed that attitude affects the implementation of technologies [27–30] and recent research has established that attitude manifests as a mediator between effort expectancy, performance expectancy, and behavioural intention [28,31]. Furthermore, the implementation climate and readiness have significant value within a work setting, as they may impact on attitudes [32–34].

Although many studies have focused on the implementation of technologies to assist people without intellectual and visual disabilities, fewer studies have focused on the implementation of technologies to assist people with intellectual and visual disabilities; especially in people with profound intellectual and multiple disabilities. Because people with profound intellectual and multiple disabilities need greater levels of support from others compared to people with a mild intellectual disability, using technologies may be challenging both for the person with profound intellectual and multiple disabilities and for the support person [35]. Both effort expectancy and attitude have been found to be related to experience and to be predictors of the intention to use technologies, while the other constructs are more focused on the performance or the organisation [27,36,37]. Since we want to focus on support people in their care for people with intellectual and visual disabilities, our study will focus on effort expectancy and attitude in relation to behavioural intention. Therefore, we set out to gain insight into the relationship between effort expectancy, attitude, and behavioural intention when implementing technologies for people with intellectual and visual disabilities. We formulated the following main research question: *What is the association of effort expectancy and attitudes of support people regarding the intention to use technologies in caring for people with intellectual and visual disabilities?*

Besides that, we wanted to explore how attitude and behavioural intention are associated with characteristics of the support people and support need levels of people with intellectual and visual disabilities. The following sub-questions were the focus of this study:

1. Which characteristics of support people are related to attitude and behavioural intention?
2. What is the effect of support need levels on attitude and behavioural intention?

### Hypotheses

We expected that both effort expectancy and attitude are predictors of the intention to use technologies [27,36,37]. However, we assumed that attitude mediates effort expectancy and that attitude is the strongest predictor of behavioural intention [28,31]. In addition, previous research showed that behavioural intention is related to age, gender, experience, and voluntariness of use [8]. Therefore, we expected that the effects of attitude on the intention to use technologies are related to support people characteristics. In addition, we expected that the effects of attitude on the intention to use technologies are related to the intensity of support needs [7].

## Materials and methods

### Research design

We used a cross-sectional quantitative design, collecting data from support people regarding the use of technologies in caring for people with intellectual disabilities (i.e. mild, moderate, or severe to profound) and visual disabilities. The questionnaire was accessible from 29 August to 26 September 2022 inclusive.

### Participants

A convenience sample of participants was selected within a Dutch healthcare organisation. This organisation supports people who are partially sighted (i.e. visual acuity is less than normal) or blind, with or without an intellectual and visual disability. Using the inclusion criteria noted below, approximately 2.500 supports people work at this healthcare organisation as healthcare professional and 30.000 are connected to the organisation as relative.

### Inclusion criteria

Relatives (i.e. family members, friends, neighbours, and volunteers) of people with intellectual and visual disabilities, and healthcare professionals (i.e., professional caregivers, allied healthcare professionals, healthcare psychologists, and teachers) who supported someone with intellectual and visual disabilities were included.

- Relatives were eligible for inclusion if they were a family member, friend, neighbour, or a volunteer helper of a person with intellectual and visual disabilities; that person needed support (i.e., intermittent, limited, and extensive to pervasive support needs); and that person was living in the facility, participated in daycare services, went to school, or participated in rehabilitation services of a healthcare organisation for people with intellectual and visual disabilities.
- Healthcare professionals were eligible for inclusion if they worked with people with intellectual and visual disabilities in one of the departments of the healthcare organisation for people with intellectual and visual disabilities (i.e. residential and daycare, rehabilitation and advice, education).
- The support needs person had both an intellectual disability as well as a visual disability.
- Participants were included if they gave their informed consent.

### Exclusion criteria

There were no clear exclusion criteria. However, participants had to be able to read Dutch and needed access to internet to fill in the questionnaire.

### Data collection

Pedagogical and Educational Sciences of the Faculty of Behavioural and Social Sciences (of the University of Groningen) approved the research plan (PED-2122-S-0079). No data that referred to a person was collected during the process. The study was conducted as a collaboration between the Academic Collaborative Centre for PIMD and a healthcare organisation for people with visual and intellectual disabilities. Participants were recruited within a healthcare organisation supporting people with intellectual and visual disabilities using digital newsletters, the intranet, social media outlets, email, and meetings. During the collection period, information was sent out by the healthcare organisation on a regular basis. Participants were informed about the project in an information letter which included a link to the questionnaire that support people could complete anonymously. Participants had to give their informed consent before they could fill in the questionnaire. These informed consent forms were saved separately from the responses to the questionnaire to guarantee the anonymity of the data.

We developed a questionnaire in collaboration with a healthcare organisation for people with intellectual and visual disabilities. We used a questionnaire that included questions about the characteristics of the support person, characteristics of the support needs person, effort expectancy, attitude, and behavioural intention. A total of 16 questions for relatives and 19 for healthcare professionals were presented in a non-randomised order; three questions on characteristics of the support person, three questions on characteristics of the support needs person, and 10 on effort expectancy, attitude, and behavioural intention for relatives; six questions on characteristics of the support person, three questions on characteristics of the support needs person, and 10 on effort expectancy, attitude, and behavioural intention for healthcare professionals.

Demographic characteristics of all support people (i.e., gender, age, and role of the support person) were gathered. In addition, healthcare professionals were asked about their position, the department in which they worked (i.e., residential and daycare, rehabilitation and advice, education), and work experience (in years). Relatives and healthcare professionals also answered self-report questions about the person they supported, concerning the latter’s intellectual disability (i.e. mild, moderate, or severe to profound), motor functioning (using the Gross Motor Function Classification System, GMFCS) [38], and auditory functioning (i.e., no hearing problems, hearing impaired, or deaf). Level of auditory functioning was included because of the prevalence of hearing loss in people with intellectual disabilities and the effect of concomitant disabilities on support needs [1,5,6,39]. Relatives answered questions about an individual with intellectual and visual disabilities, who they represented, while healthcare professionals answered questions about a group of people with intellectual and visual disabilities. Healthcare professionals who supported more than one group (i.e., mild, moderate, and severe to profound intellectual disabilities), were asked to choose the group they were most involved with or felt most connected to. It is possible that the healthcare professionals answered questions about children of relatives who were also participants in this study.

Effort expectancy, attitude, and behavioural intention were measured using a Dutch translated version of the UTAUT questionnaire [8,40]. The original questionnaire consists of eight subscales and 31 items; however, we only used the three subscales of “effort expectancy”, “attitude”, and “behavioural intention”, with a total of 10 items. The subscale of effort expectancy measures the ease of technology use [8], and we used all four items on effort expectancy from the UTAUT questionnaire. The subscale of attitude measures a person’s attitude to using technology [8]. We only used three of the four items on attitude from the UTAUT questionnaire. The item, “Working with technology is fun”, was excluded from the study because of its similarity with one of the other items. The subscale of behavioural intention consisted of three items regarding the use of technologies in the next six months. The item, “I predict I will use technology in the next six months”, was changed to, “I would like to use technology in my job in the next six months”, in order to differentiate more between items. Participants could score the items on a five-point Likert scale (i.e., (1) strongly disagree, (2) disagree, (3) neither agree nor disagree, (4) agree, and (5) strongly agree). For relatives, “working” and “in my job” were replaced by “with my family member”. Participants had to answer the questions of the UTAUT with the person/people they supported in mind. They were reminded of this before answering the questions. The items in the questionnaire can be found in Appendix A.

Adequate validity of the UTAUT model has been demonstrated in various studies, including studies focusing on healthcare technologies [41–44]. This was also performed in our own study. Content validity for the entire questionnaire was qualitatively measured [45]. We asked three healthcare professionals to fill in the questionnaire and comment on all items regarding vocabulary, order of items, scoring, ease of answering, and to reflect on possible missing items. Adjustments were made based on their comments. The item on intellectual disability was changed regarding the explanation text, and the figure of the GMFCS levels was placed above the questions rather than below. Changes to the wording of the healthcare professional’s questionnaire were also implemented for its use by relatives. Cronbach’s alpha was calculated to estimate the internal consistency of effort expectancy, attitude, and behavioural intention. Cronbach’s alpha for the four items of the effort expectancy subscale was α = .92; Cronbach’s alpha for the three items of the attitude subscale was α = .81; and Cronbach’s alpha for the three items of the behavioural intention subscale was α = .83.

### Data analysis

Data were included if a participant answered 80% of the questions (i.e. for relatives, 13 questions, and for healthcare professionals, 16 questions). Frequencies were calculated to describe the demographics of the participants. Reverse coding was used to change one negative item in the attitude subscale. We used Cronbach’s alpha to measure the reliability of the subscales and applied descriptive analyses of effort expectancy, attitude, and behavioural intention to measure the level of agreement. We also determined the correlations between effort expectancy, attitude, behavioural intention, the characteristics of the respondents, and support needs.

We categorised the characteristics of the supported person into one variable using the classification of support needs [1]. This classification uses self-report questions, on the basis of which we could derive the level of support needs (i.e., intermittent, limited, or extensive to pervasive support needs) and differentiate between people with intermittent, limited, and extensive to pervasive support needs.

We wanted to measure how effort expectancy and attitude had an effect on behavioural intention and determine whether the effect of effort expectancy was mediated through attitude. Therefore, effort expectancy was our independent variable, attitude our mediator, and behavioural intention our dependent variable. Regression analyses were performed to analyse the association between effort expectancy and attitude regarding the intention to use technologies. Firstly, we used backward stepwise regression models to determine whether the variables of gender, age, role, department, work experience, and level of support needs of the person with intellectual and visual disabilities were related to attitude and behavioural intention. The assumptions of linearity, homoscedasticity, independence, and normality were checked using a scatterplot of standardised residuals, a histogram, and the Variance Inflation Factor (VIF). Independent variables that had a significant effect (α = .05) on attitude and behavioural intention were used as covariates to build our regression model.

**Figure 1.**
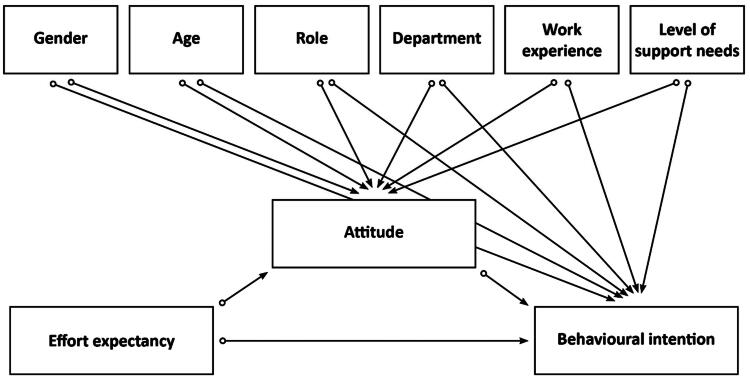
Proposed theoretical model used to measure the effects of effort expectancy, attitude, and the covariates on behavioural intention. *Note.* This figure presents effort expectancy as the independent variable, attitude as a mediator, and behavioural intention as the dependent variable. This represents the main question: *What is the association of effort expectancy and attitude of support people regarding the intention with the use of technologies for people with visual and intellectual disabilities?* Gender, age, role, department, work experience, and level of support needs are covariates related to attitude and behavioural intention. This represents the two sub-questions: (1) Which characteristics of support people are related to attitude and behavioural intention; (2) What is the effect of support needs levels on attitude and behavioural intention?

Secondly, we used regression analysis to test whether effort expectancy and attitude had an effect on behavioural intention on their own. Thirdly, a PROCESS procedure was used to test whether attitude was a mediator [46]. The PROCESS procedure measured whether there was a direct effect between effort expectancy and behavioural intention and whether there was an indirect effect of effort expectancy through attitude on behavioural intention. Figure 1 shows the proposed theoretical model used to measure the effects of effort expectancy, attitude, and the covariates on behavioural intention.

## Results

### Characteristics of the participants

A total of 313 participants took part in the questionnaire. Of these, 127 participants were excluded due to incompleteness of the questionnaire, of which 94 participants only opened the questionnaire and 33 participants answered less than 80% of the questions. After excluding these participants, we analysed the data of 186 participants. Relatives had a mean age of 60 years (*M* = 60.59; SD = 10.62) and healthcare professionals had a mean age of 45 years (*M* = 44.56; SD = 12.32). Some healthcare professionals had more than one function in the organisation. In addition, the healthcare professionals had a mean work experience of 14 years (*M* = 14.11; SD = 10.71). More descriptive statistics about the characteristics of the support people can be found in Table 1.

**Table 1.** Characteristics of the support people.

	Relatives		Healthcare professionals	
	n	%	n	%
Subsample size	58		128	
Gender				
Female	41	71	99	88
Male	15	26	11	10
Role				
Family members	55	95		
Informal contacts	3	5		
Professional caregivers			92	72
Allied healthcare professionals			22	17
Healthcare psychologists			7	5
Teachers			12	9
Department				
Living and daycare			98	77
Rehabilitation and advice			15	12
Education			15	12

*Note.* Gender is not 100% because of missing data. Some healthcare professionals had more than one function in the organisation, therefore the sum of Role and Department is more than 100%.

All participants supported someone who was severely partially sighted to blind and had a mild to profound intellectual disability. According to these participants, 53.1% (*n* = 43) had a severe to profound intellectual disability and a severe to profound motor disability; 37.1% (*n* = 26) of all people with a moderate intellectual disability had a mild motor disability and 37.1% (*n* = 26) had a moderate motor disability; while 57.1% (*n* = 20) of all the people with a mild intellectual disability had a mild motor disability. Descriptive statistics and the classifications of support needs are reported in Table 2.

**Table 2.** Characteristics of the people with support needs.

	People with support needs supported by relatives		People with support needs supported by healthcare professionals	
	n	%	n	%
Subsample size	58		128	
Level of intellectual disability				
Mild	14	24	21	16
Moderate	24	41	46	36
Severe to profound	20	35	61	48
Level of gross motor functioning (GMFCS)				
No motor problems	11	19	9	7
II	20	35	39	31
III	14	24	38	30
IV	8	14	24	19
V	5	9	18	14
Auditory functioning				
No hearing loss	46	79	55	43
Hearing impaired	10	17	64	50
Deafness	2	3	9	7
Level of support needs*				
Intermittent	14	24	21	16
Limited	19	33	39	31
Extensive to pervasive	25	34	68	53

*Note.* *The level of support needs was derived by using the level of intellectual functioning, the level of motor functioning, and the level of auditory functioning [1,5,6].

### Agreement of effort expectancy, attitude, and behavioural intention

**Figure 2.**
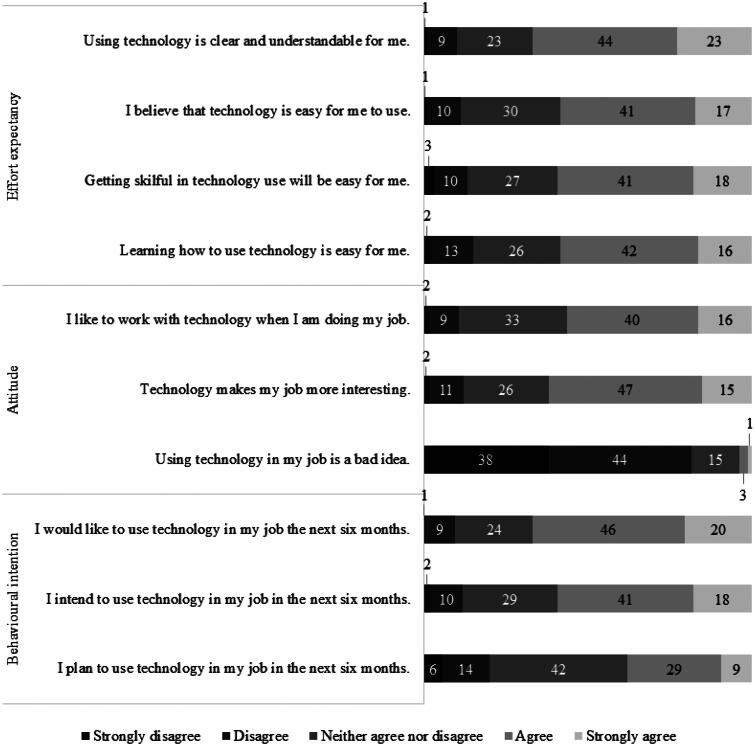
Scores on items of effort expectancy, attitude, and behavioural intention of participants (*n* = 186) in percentages.

Scores on all items can be found in Figure 2. In general, participants agreed with items on effort expectancy, attitude, and behavioural intention. They especially agreed with the item, “Using technology is clear and understandable for me.”, where 67% agreed and 10% disagreed. Participants also agreed with the item, “I would like to use technology in my job in the next six months.”, with 66% agreeing and 10% disagreeing. The item most participants (82%) disagreed on was, “Using technology in my job is a bad idea.”. Only 4% agreed with this item.

### Correlations between effort expectancy, attitude, behavioural intention, and the covariates

For healthcare professionals, there were moderate positive correlations between the characteristics of age and experience (*r* = .60; *p* < .001). For relatives, there was a moderate negative correlation between the construct of effort expectancy and the characteristic of age (r = −0.47; *p* < .001). Furthermore, there were correlations between the constructs. A strong positive association was found between attitude and behavioural intention for both relatives (*r* = .78; *p* < .001) and healthcare professionals (*r* = .71; *p* < .001). For healthcare professionals, there were moderate positive correlations between effort expectancy and attitude (*r* = .62; *p* < .001), and effort expectancy and behavioural intention (*r* = .45; *p* < .001). The results of the correlation analysis are summarised in Table 3.

**Table 3.** Correlations between effort expectancy, attitude, behavioural intention, and the covariates in relatives and healthcare professionals.

		Gender	Age	Support needs	Department	Experience	EE	Attitude	BI
Relatives	Gender	1							
	Age	−0.10	1						
	Support needs	−0.05	−0.18	1					
	EE	−0.24	−0.05	.25			1		
	Attitude	−0.06	−0.21	.12			.17	1	
	BI	−0.02	−0.17	.10			.23	.78**	1
Professionals	Gender	1							
	Age	−0.03	1						
	Support needs	.13	−0.03	1					
	Department	.03	.04	.04	1				
	Experience	−0.06	.60**	−0.05	−0.07	1			
	EE	−0.15	−0.47**	−0.12	−0.14	−0.31**	1		
	Attitude	−0.09	−0.19*	−0.15	−0.02	−0.16	.62**	1	
	BI	−0.01	−0.14	−0.07	.11	−0.15	.45**	.71**	1

*Note.* EE = effort expectancy; BI = behavioural intention; experience = work experience intellectual = level of intellectual functioning; motor = level of motor functioning; and auditory = level of auditory functioning; *correlation is significant at level .05; **correlation is significant at level .01.

### Variables related to attitude

The assumptions were checked before conducting a regression analysis. The scatterplot of standardised residuals, the histogram, and the VIF showed that the data met the assumptions for linear regression. The backward stepwise regression method indicated a significant result for age (t(178) = −3.82; *p* < .001), which means that younger relatives and healthcare professionals scored higher on attitude compared to older relatives and healthcare professionals. Age explained 7.1% of the variance in attitude (R^2^ *Adjusted* = .07, F(1, 177) = 14.62, *p* < .001).

For healthcare professionals, work experience (t(122) = −0.62; *p* = .476) and department (t(122) = −0.75; *p* = .453) did not have a significant effect on attitude.

### Variables related to behavioural intention

The assumptions for linear regression were met according to the scatterplot of standardised residuals, the histogram, and the VIF. There was only a significant effect for age (t(178) = −2.96; *p* = .004). Data indicated that younger relatives and healthcare professionals scored higher on behavioural intention than older relatives and healthcare professionals. Age explained 4.2% of the variance in behavioural intention (R^2^ *Adjusted* = .04, F(1, 177) = 8.74, *p* = .004).

The backward stepwise regression model showed that there was no significant effect for work experience (t(122) = .-0.72; *p* = .475) or department (t(122) = 1.31 = 8; *p* = .171) on behavioural intention.

### Mediating effect of attitude on behavioural intention

**Figure 3.**
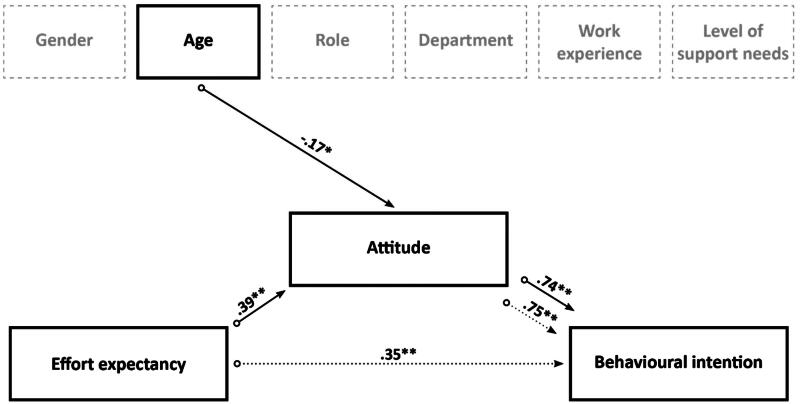
Effects of effort expectancy, attitude, and the covariates on behavioural intention. *Note.* This figure shows behavioural intention as the dependent variable, effort expectancy as the independent variable, attitude as a mediator, and age as a covariate. The β values and their level of significance are displayed; *p < .05, **p < .001). The dotted lines represent two models: (1) the effect of effort expectancy on behavioural intention without attitude and age, (2) the effect of attitude on behavioural intention without effort expectancy and age. The straight lines represent the third model, where effort expectancy, attitude, and age are taken into account.

The effects of effort expectancy on behavioural intention and of attitude on behavioural intention were represented in two models. In the first model, the results indicated that effort expectancy explained 12.1% of the variance (R^2^ = .12, F(1, 184) = 25.38, *p* < .001) and that it had a significant effect on behavioural intention (β = .35; t(185) = 5.04; *p* < .001). In the second model, attitude had a significant effect on behavioural intention (β = .75; t(185) = 15.55; *p* < .001) and explained 56.8% of the variance (R^2^ = .57, F(1, 184) = 241.81, *p* < .001). The results of both models are presented in Figure 3.

While Figure 1 represents the proposed theoretical model with all covariates included, the results showed that only age had an effect on attitude and behavioural intention. Therefore, we only included age in Model 3, with behavioural intention as a dependent variable, effort expectancy as an independent variable, attitude as a mediator, and age as a covariate. Further investigation with the PROCESS procedure indicated that effort expectancy, attitude, and age explained 57.3% of the variance (R^2^ = .57, F(3, 176) = 78.61, *p* < .001). Effort expectancy had a significant effect on attitude (β = .39; t(178) = 5.56; *p* < .001) and age also had a significant effect on attitude (β = −0.17; t(178) = −2.49; *p* = .014). Attitude had a significant effect on behavioural intention (β = .74; t(177) = 13.28; *p* < .001). The results showed that the direct effect of effort expectancy on behavioural intention was not significant (β = .04; t(177) = .74; *p* = .462), but that the indirect effects of effort expectancy on behavioural intention could be explained *via* attitude (95% CI: 146; .382), confirming attitude as having a mediation effect. Figure 3 presents the results obtained from the PROCESS procedure.

## Discussion

### Main findings

The purpose of this study was to gain insight into the association of effort expectancy and attitude of support people (i.e. relatives and healthcare professionals) regarding the intention to use technologies in care for people with intellectual and visual disabilities. The relationship of attitude and behavioural intention with support person’s characteristics and the level of support needs was also analysed. The results indicated that the attitude of the support person had a direct effect on their behavioural intention to use technology in support. The effect of effort expectancy on behavioural intention was mediated through attitude, while the direct effect of effort expectancy was not significant. Younger support people had a more positive attitude compared to older support people; however, age did not affect behavioural intention when attitude was also included. In addition, there was no relationship between attitude, behavioural intention, and level of support needs.

### Theoretical reflections

Based on previous studies, we expected the characteristics of support people to be related to attitude and behavioural intention [8]. However, we found that only the age of the support person was related to attitude and behavioural intention, while gender, role, department, and work experience were not related to either attitude or behavioural intention. Further analysis indicated that the age of the support person affects attitude, but that age does not affect the intention to use technologies when attitude is also considered. Thus, the difference in attitude between younger and older support people affects the intention to use technology and not age itself. Therefore, focus should be on creating positive attitudes to technology use in older support people by creating an environment where they can learn from younger support people and where they can experiment [30].

In addition, our study showed that the gender of the support person was not related to the intention to use technology in people with support needs. Although gender may have an impact in certain cultures [47], the attitudes of men and women are generally positive and the use of technologies is the same among men and women [48,49]. This outcome contrasts with that of (8], who found that gender did have an effect. The use of technology might have shown differences between men and women when the UTAUT was designed; however, today digital skills in men and women are more or less the same in European countries [50], and older people also feel more comfortable using technology [51].

According to previous research [7], we expected that the level of support needs would be related to attitude and behavioural intention. However, the findings of the current study did not support this assumption. Although people with intellectual and visual disabilities may require support when using technologies, the level of support needs did not affect the attitude and behavioural intention of support people in our study [52] also showed that attitudes of support people were not affected by the level of support needs. This discrepancy might be attributed to the fact that the intention to use technology is related, but does not necessarily lead to the actual use of technologies [53]. Work-related pressure and stress in support people might affect the actual use of technology [54,55]. While we observed that the attitude of support people was generally positive, there may be other barriers that prevent sustainable implementation of technology.

Access to technology is only reserved for a select group of people with support needs [56], and more accessible types of technologies, such as mainstream technologies, are used less in people with severe support needs [16]. During the COVID-19 pandemic, it became very clear that people with support needs were not included in the digital world and, despite the best efforts at the beginning of the pandemic, digital contact was challenging and less customary in people with more severe support needs [7,57]. Thus, the benefits of technology do not seem to be readily accessible to those with more severe support needs, as they appear to be overlooked when implementing them. Barriers to the use of technologies seem to be related primarily to not understanding the purpose of the technology, misconceptions about the technologies, and not perceiving the use of technologies as part of the care [58].

Based on previous research, we expected effort expectancy and attitude to be related to behavioural intention [8,27–30]. The results of our study confirmed this assumption. In addition, studies have shown that attitude manifests itself as a mediator between effort expectancy and behavioural intention [28,31]. In our study, attitude was the highest predictor of behavioural intention and mediated the effect of effort expectancy and behavioural intention. The attitude of support people appears to be the key element that needs to be addressed when implementing technologies for people with support needs.

The attitude of support people may be influenced by factors such as knowledge, skills, experience, perceived enjoyment, opinions of others, and trust in technology [30,59–62]. For example, it has been found that more knowledgeable students had a more positive attitude to e-learning compared to less knowledgeable students [63]. In addition, paying attention to the dimension of communication has been found to be important, as people with support needs may require additional support when using technologies and therefore must somehow communicate these requirements [64]. Furthermore, context and culture also play significant roles in the way support people consider those with support needs and how they perceive their development and the ability to use technologies [64–66]. Therefore, paying attention to these factors could change a negative attitude or resistance to the use of technologies into a more positive attitude [29] and thus be an important step towards desired change [52].

### Methodological reflections

The findings of this study may contribute to our understanding of how effort expectancy and attitude of support people have an effect on the intention to use technologies in care for people with intellectual and visual disabilities. However, when interpreting the results, we need to take a number of aspects into account. Although the response was high, there was an imbalance in the kind of participants and their characteristics, with more healthcare professionals participating compared to relatives and more women compared to men. In addition, relatives in our study were generally older compared to the professionals. Therefore, although the results for the relatives suggest that they are less positive than healthcare professionals about the use of technologies in caring for people with support needs, this may have to do with age rather than their relationship with the person with support needs. Although the response was high, we did not reach all possible participants. One hundred twenty-eight of the healthcare professionals (N ≈ 2.500) and 58 of the relatives (N ≈ 30.000) responded. The convenience sample was used to reach a broad audience. However, this increases the risk of selection bias.

Besides that, we should pay attention to the way the constructs were measured; especially effort expectancy since it might be less related to the use of technologies in collaboration to people with support needs. Although participants had to answer the questions with one person or a group of people with support needs in mind, it might be difficult to answer these questions and relate this to using technology with people with support needs. This may have affected the validity of the results. However, we did use an existing instrument with adequate validity that can also be used by healthcare professionals [8,40].

In addition, participants were recruited within an organisation caring for people with a visual disability, with or without an intellectual disability. Although the prevalence of visual impairments is generally high among people with profound intellectual disabilities [2–4], this is not the case for people with mild or moderate intellectual disabilities. Therefore, we question whether the results can be generalised to people with mild or moderate intellectual disabilities and whether they apply to all organisations. Visual impairments, challenges in processing sensory information, and blindness are highly prevalent in people with profound intellectual disabilities but less in people with mild intellectual disabilities [2–4]. Therefore, generalisation to people with profound intellectual disabilities may be higher compared to people with mild intellectual disabilities. Besides that, we developed the questionnaire in collaboration with healthcare professionals. We did not involve relatives in the development of the questionnaire due to high demands enforced on relatives. However, they were invited to discuss the results and were sent an easily readable infographic with the results.

The length of a questionnaire may affect its reliability, with long questionnaires found to be related to loss of interest and unreliable answers [67]. Consequently, we were not able to include all of the items (31) of the UTAUT. This also limited the number of characteristics of the support people we could ask. For example, education level, experience with technologies, and nature of a disability could influence the use of technologies [8,68]. Even so, some participants answered less than 80% of the questions, which could lead to response bias, as we used a non-randomised questionnaire and the same questions towards the end of the questionnaire were most frequently missed. Although we limited the number of questions, the questionnaire might still have been too long.

### Recommendations for future research

Future research on the current topic is recommended. In this study, we only analysed the relationship between effort expectancy, attitude, and behavioural intention of support people. However, there are many factors that can influence the process of implementation of technologies. Therefore, further research should focus on the implementation process and the factors that influence this implementation. In our study, we focused on individual factors such as effort expectancy, attitude, and behavioural intention. However, factors such as confidence, motivation, knowledge, and self-efficacy are also related to implementation [69,70]. Moreover, in addition to individual factors, there are also organisational factors, environmental factors, intervention factors, and factors related to the person with support needs. Organisational factors include implementation climate, readiness for implementation, awareness, staff turnover, leadership, and bureaucracy [69,71]. Environmental factors include external support, training, collaboration, external policy, resources, and community demands [71–73]. Examples of intervention factors are complexity, adaptability, effectiveness, and efficiency [69–71]. Factors related to the person with support needs include motivation, engagement, and physical situation [69,71].

Some of these factors may be modifiable, while others might be difficult to change or not be flexible at all. However, there is a lack of knowledge about these factors specifically concerning the use of technology in people with support needs. Hence, it is imperative for future studies to gain more understanding of the variables that can be manipulated and their impact on the use of technologies in care for people with support needs. In addition, it would be interesting to determine which factors are generic in all implementation processes and which factors are technology specific. While it is plausible that universal factors apply to all forms of implementation of innovative care practices, the implementation of new technology may also involve specific factors.

### Recommendations for practice

This study has identified how effort expectancy, attitude, and behavioural intention of support people are related when using technologies to assist people with support needs. These findings provide insights relevant to healthcare organisations that may be implementing technologies for people with support needs. In addition, the results of this study indicate that the attitude of support people is a key element in the implementation of technologies in people with support needs. Therefore, it may be important for healthcare organisations to pay attention to the attitudes of support people. Such attitudes may be influenced by many factors, including knowledge [60,63], which could help support people improve their technological skills. Developing technological skills and creating a positive attitude towards technologies may help support people to better use technologies in caring for people with support needs [74,75]. Therefore, education about how to use these technologies, the effects on people with intellectual and visual disabilities, and a safe space to experience the manifold possibilities of the use of technology may facilitate implementation [26,76].

## Conclusions

The findings of this study provide a new understanding of some of the factors involved in the implementation of technologies in the care of people with support needs. Support people play a significant role in this implementation and, therefore, their effort expectancy and attitude towards the use of technologies is important. The findings showed that effort expectancy and attitude have an effect on the intention to use technologies in care for people with support needs, but that attitude is the key element in predicting the intention to use technologies.

## Supplementary Material

Appendix_A_SuppInfo.docx

## Data Availability

The data that support the findings of this study are available from the corresponding author, Lotte Piekema, upon request.
