# Supplementary material for: Attitudes of support people: a key element when implementing technologies for people with intellectual and visual disabilities
Source: Disabil Rehabil Assist Technol. 2024 Aug 8;20(2):432–43. doi: 10.1080/17483107.2024.2387774 (PMC11789705; doi:10.1080/17483107.2024.2387774)
Supplement: Appendix_A_SuppInfo.docx [file IIDT_A_2387774_SM6533.docx]

**Appendix A**

| **Variables** | **Items on** | **Answer options** |
| --- | --- | --- |
| Support person | 1. Gender | Male/Female/Other |
|  | 1. Age | Open answer |
|  | 1. Role of support person | Relative/volunteer, friend, neighbour/healthcare professional |
| Healthcare professional job | 1. Job title      1. Department 2. Work experience | Residential counsellor/daycare supervisor/coordinating supervisor/ teacher/physiotherapist/occupational therapist/speech therapist/ behavioural scientist/manager/open answer  Residential and daycare/Rehabilitation and advice/Education  Open answer |
| Person with support needs | 1. Level of intellectual disability | Mild/moderate/severe to profound |
|  | 1. Level of gross motor problems | No/GMFC level 2/GMFC level 3/GMFC level 4/GMFC level 5 |
|  | 1. Level of hearing impairment | No/hearing impairment/deaf |
| Effort expectancy | 1. Using technology is clear and understandable for me. | Likert scale: (1) strongly disagree, (2) disagree, (3) neither agree nor disagree, (4) agree, and (5) strongly agree. |
|  | 1. It would be easy for me to become skilful at using technology. | Likert scale: (1) strongly disagree, (2) disagree, (3) neither agree nor disagree, (4) agree, and (5) strongly agree. |
|  | 1. I believe that technology is easy for me to use. | Likert scale: (1) strongly disagree, (2) disagree, (3) neither agree nor disagree, (4) agree, and (5) strongly agree. |
|  | 1. Learning how to use technology is easy for me. | Likert scale: (1) strongly disagree, (2) disagree, (3) neither agree nor disagree, (4) agree, and (5) strongly agree. |
| Attitude | 1. Using technology in my job is a bad idea. | Likert scale: (1) strongly disagree, (2) disagree, (3) neither agree nor disagree, (4) agree, and (5) strongly agree. |
|  | 1. Technology makes my job more interesting. | Likert scale: (1) strongly disagree, (2) disagree, (3) neither agree nor disagree, (4) agree, and (5) strongly agree. |
|  | 1. Working with technology is fun. | Likert scale: (1) strongly disagree, (2) disagree, (3) neither agree nor disagree, (4) agree, and (5) strongly agree. |
| Behavioural intention | 1. I intend to use technology in my job in the next six months. | Likert scale: (1) strongly disagree, (2) disagree, (3) neither agree nor disagree, (4) agree, and (5) strongly agree. |
|  | 1. I would like to use technology in my job the next six months. | Likert scale: (1) strongly disagree, (2) disagree, (3) neither agree nor disagree, (4) agree, and (5) strongly agree. |
|  | 1. I plan to use technology in my job in the next six months. | Likert scale: (1) strongly disagree, (2) disagree, (3) neither agree nor disagree, (4) agree, and (5) strongly agree. |
